# Cutis Laxa

**Published:** 1890-03-08

**Authors:** 


### CUTIS LAXA.

Dr. Otto Seifert, of Wurzburg, with his assistant, Dr. Du Mesnil, have shown that the curious condition known as *cutis laxa*, in which the skin can be drawn out like a sheet of indiarubber, is not owing to any actual increase in the proportion of the elastic fibres, but to the transformation of the true cutis into a homogeneous structureless myxomatous layer, from which the fasciculæ of connective tissue fibres, &c., which should have counteracted the extensibility of the yellow, had entirely vanished.

